# Developmental Loci Harbor Clusters of Accelerated Regions That Evolved Independently in Ape Lineages

**DOI:** 10.1093/molbev/msy109

**Published:** 2018-06-18

**Authors:** Dennis Kostka, Alisha K Holloway, Katherine S Pollard

**Affiliations:** 1 Departments of Developmental Biology and Computational & Systems Biology, Pittsburgh Center for Evolutionary Biology and Medicine, University of Pittsburgh School of Medicine, Pittsburgh, PA; 2 Gladstone Institutes, San Francisco, CA; 3 Phylos Bioscience, Portland, OR; 4 Department of Epidemiology & Biostatistics, Institute for Human Genetics, Quantitative Biology Institutes, and Institute for Computational Health Sciences, University of California, San Francisco, CA; 5 Chan-Zuckerberg Biohub, San Francisco, CA

**Keywords:** positive selection, biased gene conversion, likelihood ratio test, enhancer, development, primates

## Abstract

Some of the fastest evolving regions of the human genome are conserved noncoding elements with many human-specific DNA substitutions. These human accelerated regions (HARs) are enriched nearby regulatory genes, and several HARs function as developmental enhancers. To investigate if this evolutionary signature is unique to humans, we quantified evidence of accelerated substitutions in conserved genomic elements across multiple lineages and applied this approach simultaneously to the genomes of five apes: human, chimpanzee, gorilla, orangutan, and gibbon. We find roughly similar numbers and genomic distributions of lineage-specific accelerated regions (linARs) in all five apes. In particular, apes share an enrichment of linARs in regulatory DNA nearby genes involved in development, especially transcription factors and other regulators. Many developmental loci harbor clusters of nonoverlapping linARs from multiple apes, suggesting that accelerated evolution in each species affected distinct regulatory elements that control a shared set of developmental pathways. Our statistical tests distinguish between GC-biased and unbiased accelerated substitution rates, allowing us to quantify the roles of different evolutionary forces in creating linARs. We find evidence of GC-biased gene conversion in each ape, but unbiased acceleration consistent with positive selection or loss of constraint is more common in all five lineages. It therefore appears that similar evolutionary processes created independent accelerated regions in the genomes of different apes, and that these lineage-specific changes to conserved noncoding sequences may have differentially altered expression of a core set of developmental genes across ape evolution.

## Introduction

Accelerated sequence evolution is a hallmark of both positive selection and loss of constraint. Therefore, the comparative genomic signature of sequence change in a lineage of interest compared with conservation in other lineages has been used to identify genome sequences that are candidates for explaining evolution of lineage-specific traits. The requirement of conservation in other lineages serves two purposes. First, it suggests functional constraint, which enables genome-wide scans to focus on regions where accelerated evolution is most likely to have meaningful consequences. This is particularly helpful for discovering accelerated noncoding elements without clear-cut functional annotations, and less so for tests of accelerated evolution in well-annotated genes (Kosiol, Vinar, et al. 2008). The second reason to focus on regions that are otherwise conserved is the higher power to detect a shift in evolutionary rate, including shifts from conserved to neutrally evolving (e.g., loss of function) or weak positive selection (Pollard, Salama, et al. 2006).

This approach was applied genome-wide to identify human accelerated regions (HARs) that experienced significantly more substitutions than expected in the human lineage since divergence from the common ancestor with chimpanzees (Pollard, Salama, et al. 2006; Prabhakar, Noonan, et al. 2006; Bird, Stranger, et al. 2007, Bush and Lahn 2008). It has also been applied to other lineages, including fruit flies (Holloway, Begun, et al. 2008), several vertebrates (Ferris, Abegglen, et al. 2018), the common ancestor of therian mammals (Holloway, Bruneau, et al. 2016), and the common ancestor of bats (Booker, Friedrich, et al. 2016). The ∼2700 HARs identified to date are mostly noncoding, and many have epigenomic signatures suggestive of enhancer function in human cells. To date, 62 out of 92 HARs that were prioritized based on evidence of a regulatory role (67.4%) showed enhancer activity in transient transgenic reporter assays in mouse or fish embryos, and seven HARs are known to drive different expression patterns with the human compared with orthologous chimpanzee sequence (Hubisz and Pollard 2014). Consistent with this role in developmental gene expression, HARs are enriched in genomic loci with transcription factors and other genes involved in regulation of development (Capra, Erwin, et al. 2013, Kamm, Pisciottano, et al. 2013, Gittelman, Hun, et al. 2015). HARs also occur at a higher rate at the distal ends of chromosomes, where sequence divergence is generally higher (Pollard, Salama, et al. 2006).

We were curious if the prevalence of lineage-specific accelerated regions (linARs) and their association with developmental gene regulation is unique to humans, or if chimpanzees and other primates share this genomic feature. Previous work identified linARs that are accelerated across primates as a clade (i.e., with substitutions in multiple primate lineages), and these were indeed mostly noncoding and associated with developmental loci (Lindblad-Toh, Garber et al. 2011). With more genomes now available, it is possible to query each primate lineage individually for linARs and to compare patterns across species. To do so requires a statistically rigorous method for assessing evidence of acceleration in each lineage for a consistent set of conserved genomic regions. We solved this problem by implementing a model selection procedure that uses likelihood ratio statistics to evaluate support in the multiple sequence alignment of a given genomic region for a set of models, each with combinations of acceleration or no acceleration in different lineages. By standardizing data and methods across species, we help to address some of the reasons that previously published lists of HARs have low overlap (Franchini and Pollard 2017, Levchenko, Kanapin, et al. 2018), namely that each study used different alignments, bioinformatics filters, definitions of conservation, and definitions of acceleration.

In addition to testing for acceleration, our model selection procedure also enables evaluation of evidence for GC-biased gene conversion (gBGC) in different lineages. gBGC is a recombination associated process that mimics positive selection by increasing the rate of fixation of GC alleles, whereas decreasing the rate of fixation of AT alleles. Previous analyses of HARs found evidence of gBGC, especially in the fastest evolving HARs and in regions of high recombination in modern humans (Pollard, Salama, et al. 2006, Galtier and Duret 2007, Lindblad-Toh, Garber, et al. 2011, Kostka, Hubisz, et al. 2012). Using the weak-mutation model of Kostka et al. (Kostka, Hubisz, et al. 2012), our method therefore evaluates each conserved genomic region with a collection of models that includes all combinations of unbiased acceleration (loss of constraint or positive selection), gBGC, or no acceleration in each lineage.

We applied this approach to whole-genome alignments and identified 5916 conserved elements with evidence of accelerated substitutions in at least one of five apes: human (*Homo sapiens*), chimpanzee (*Pan troglodytes*), gorilla (*Gorilla gorilla gorilla*), orangutan (*Pongo pygmaeus abelii*), and gibbon (*Nomascus leucogenys*). These ape linARs are roughly equal in number across species, mostly noncoding, and enriched nearby genes that are involved in regulation of development. Interestingly, a number of developmental loci harbor distinct linARs that are accelerated in different apes, suggesting that shared developmental pathways have experienced independent bursts of regulatory evolution across distinct ape lineages. Unbiased acceleration, consistent with positive selection or loss of strong constraint, is more prevalent than gBGC in all species, although each ape has a substantial minority of linARs (approximately one quarter) that are GC biased. These findings clarify that linARs are not a human-specific phenomenon.

## New Approaches

### Testing for Lineage-Specific Acceleration across Multiple Lineages

We developed a statistical procedure to test a multiple sequence alignment for evidence of accelerated substitution rate in any combination of lineages in a phylogenetic tree. Our method is based on contrasting a suitably chosen phylogenetic null model with a series of alternative models using likelihood ratio tests (LRTs). Briefly, let *ϑ* denote the full set of parameters of a phylogenetic model, and let *ϑ*_0_ denote the subset of *ϑ* that is optimized to fit the null model. In the following, we will identify models with their respective parameter sets and use these terms interchangeably. We then construct a set of alternative models that code for lineage specific substitution rate acceleration as follows: Each lineage (we focus on sets of leaf-branches in the phylogenetic tree of *ϑ*, but internal branches could be used as well) is endowed with two parameters in addition to *ϑ*_0_: a parameter *S*_l_* *≥ 0 for modeling an unbiased substitution rate increase, and a parameter *B*_l_* *≥ 0 for modeling a GC-biased substitution rate increase (l denotes the lineage/branch). Modeling GC-biased acceleration allows us to detect elements that are accelerated due to gBGC and mismatch repair (Pollard, Salama, et al. 2006, Galtier and Duret 2007, Lindblad-Toh, Garber, et al. 2011, Kostka, Hubisz, et al. 2012), whereas unbiased acceleration captures loss of constraint and positive selection. In short, the parameter *S* codes for a selection coefficient and *B* codes for a gene conversion disparity in a population genetic model, and both are related to the rate matrix of a phylogenetic model via eventual fixation probabilities (see Kostka, Hubisz, et al. 2012). The null model is then the special case where both these parameters are constrained to zero in all lineages (no GC-biased or unbiased acceleration). Each alternative model is then characterized by “activating” combinations of *S* and *B* across lineages; more precisely, an alternative model *ϑ*_A_ is defined as *ϑ*_A_ = *ϑ*_0_ ∪ {*S_j_*}*_j_*_∈__*K*_ ∪ {*B_i_*}_*i*__∈__*M*_ where *K* denotes the set of lineages with *S* not constrained to zero and *M* the set of lineages with *B* not constrained to zero. Studying *n* lineages there exist 2^2^^*n*^ such models (including the null model), and a model *ϑ_i_* is nested in another model *ϑ_j_* when it is a subset (*ϑ_i_* ⊂ *ϑ_i_*). To compare nested models, we employ LRTs that quantify the amount of support in the alignment data for the more complex model in the comparison, taking model complexity into account. However, comparing all alternative models to the null model exhaustively quickly becomes computationally demanding; in addition, with this approach we learn nothing about how different alternative models compare with each other.

### An Efficient Model Selection Algorithm

**Fig. 1.**
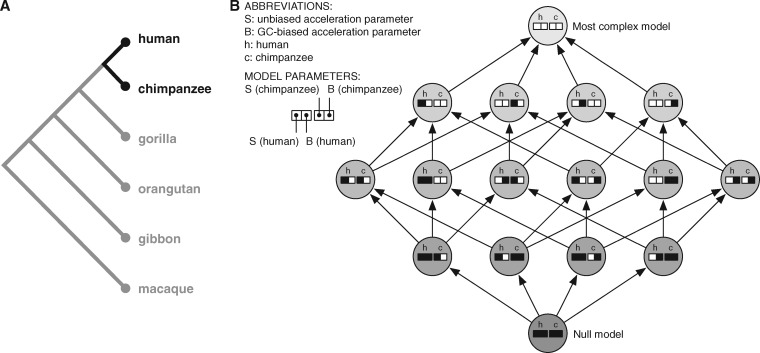
Testing for acceleration in five apes. To test a conserved noncoding element for an accelerated rate of unbiased and/or GC-biased substitutions in any of five apes, we perform a series of up to 1024 nested LRTs. (*A*) The apes tested and their phylogenetic relationships (branches not to scale). For illustrating the approach, consider the possible models for just the human and chimpanzee branches. (*B*) Example model selection procedure for two species. The null model (bottom node) has no unbiased acceleration (*S* = 0 on both human and chimpanzee branches) and no GC-biased acceleration (*B* = 0 on both branches). There are four models with one of the parameters not equal to zero (next set of four nodes): *S* > 0 in human (right-most), *S* > 0 in chimpanzee (second from right), *B* > 0 in human (second from left), and *B* > 0 in chimp (left-most). These are nested inside the six (i.e., 4 choose 2) possible models with two parameters constrained to zero, which are nested inside the four possible models with one parameter constrained to zero. The full model (top node) has all four parameters not constrained to zero. Our algorithm starts with the null model and performs the LRT corresponding to each subsequent arrow moving from the bottom to the top of the graph of possible models, stopping if none of the models with an additional parameter greater than zero has significantly higher likelihood than the most complex model visited. For all five primates, the graph of nested models has 1024 nodes and a similar structure.

To address these limitations of exhaustive model comparisons, we designed a forward model selection algorithm that takes advantage of the hierarchical structure present in a set of alternative models. Specifically, if we assume the null model at the bottom, we can start to build up a hierarchy of models by connecting it with all models having one extra free parameter. That is, the set of models {*ϑ_i_* such that | *ϑ_i_* ∖ *ϑ*_0_ | = 1} form the next level, where *i* indexes different alternative models, | ⋅ | denotes the cardinality of a set, and ∖ the set difference. Iterating this approach, we recursively build up a directed acyclic graph (DAG) with models as nodes and edges connecting each model to all other models with one extra free parameter (see fig. 1 for an example).

For model selection, we then traverse the edges in this DAG breadth-first, performing a LRT for each edge, and annotate the child node of the comparison (i.e., the model with one extra parameter) with the LRT *P*-value. If the child node is already annotated with a *P*-value (from an earlier comparison, typically the nodes in the model DAG have more than one child), we update this annotation to reflect the maximum of the already annotated and the current *P*-value. This *P*-value annotation allows us to implement an *early stopping criterion*: We only perform comparisons if (1) the parent node of the comparison is the null model or (2) the parent node of the comparison was already part of a comparison and the corresponding LRT *P*-value was smaller than a predefined cut-off *P*_cut_. In this way, we stop model assessment (i.e., DAG traversal) early if the alignment data do not support more complex phylogenetic models.

This series of model comparisons produces a set of alternative models with evidence for lineage-specific substitution rate acceleration (i.e., all examined models with *P*-values less than *P*_cut_). Importantly, all identified models have also been tested versus models nested within them (parents). Finally, we annotate the underlying alignment with a “best-fitting” model as follows: If all models with *P*-values less than *P*_cut_ are nested within each other (i.e., they form a path in the DAG), we annotate the alignment with the most complex model. If nonnested models are recovered, we collect all the models that have no recovered children and perform model selection with the Akaike Information Criterion (AIC). Like the LRT the AIC reflects a trade-off between model complexity and model fit, but in contrast to the LRT it can be applied to situations where the models under consideration are not nested. Overall, this procedure outputs a single model per alignment block, which is either the null model or the best alternative model. If an alternative model is chosen, this represents a combination of acceleration (unbiased or GC-biased) across lineages, which is significantly more likely than the phylogenetic null model given the alignment data. Additional details are provided in the Materials and Methods and supplementary text, Supplementary Material online.

### Implementation

The model selection method is implemented as an open-source software package in **R**, called linACC (code available at http://www.kostkalab.net/software.html, last accessed June 1, 2018 and at https://omictools.com/linacc-tool, last accessed June 1, 2018). The package is based on methods implemented in the RPHAST package (Hubisz, Pollard, et al. 2011).

### Simulation Study

We performed a simulation experiment to assess our approach. We used the phylogenetic neutral model underlying the 100-way most-conserved track from UCSC. We then re-scaled the tree by a factor of 0.1 to reflect the selective constraint in the phastCons elements analyzed below. Next, we simulated data with unbiased acceleration on the human branch or on both the human and gorilla branch. The strength of acceleration was either zero (no effect, for assessing the false positive rate) or corresponding to 1, 3, 5, or 10 expected substitutions per 100 base pairs (bp) along the branch with acceleration. For comparison, under neutral evolution we expect about 0.6 substitutions per 100 bp on the human branch and 0.9 on the gorilla branch. On the other end of the spectrum, the most accelerated HARs show on the order of 10 substitutions per 100 bp on the human branch. Thus, the simulation covers a reasonable range of substitution rates. For each combination of parameters, we simulated 100 alignments of 100-bp length and applied linACC to perform model selection and testing. The results show that the proposed model selection procedure can detect acceleration on multiple branches with 100-bp alignment blocks (table 1). When acceleration is moderate (<5), the lineage with acceleration is sometimes inferred incorrectly, even though acceleration is correctly detected. The average phastCons element we analyze below is 148.8 bp long, so we expect these simulations to conservatively estimate power and lineage accuracy in this application.

**Table 1.** Results of Simulations with Acceleration on the Human Lineage or on the Human and Gorilla Lineages.

	No. of Expected Substitutions		Percentage Alignments Annotated with acceleration in				
	Human	Gorilla	Human	Gorilla	Human and Gorilla	Other Primates	No Acceleration
**Human acceleration**	NA	NA	0	1	0	7	**92**
	1	NA	21	0	0	15	**64**
	3	NA	**64**	0	0	16	20
	5	NA	**92**	0	1	6	2
	10	NA	**96**	0	1	3	0
**Human and gorilla acceleration**	3	3	20	17	**48**	12	3
	5	3	19	0	**66**	15	0
	10	3	15	0	**74**	11	0
	3	5	3	8	**73**	15	1
	5	5	2	7	**75**	17	0
	10	5	6	0	**86**	8	0
	3	10	0	13	**78**	9	0
	5	10	0	2	**90**	8	0
	10	10	0	0	**87**	13	0

The first row corresponds to data generated by the null model and shows the false positive rate; that is, the percentage of alignments annotated to an acceleration column, even though they were generated by the null model. Expected substitutions show the level of acceleration simulated. NA, No acceleration, close to zero expected substitutions per 100 bp. Bold text indicates the correct inference (i.e., lineage with simulated acceleration or no acceleration).

## Results

### Apes Have Lineage-Specific Accelerated Regions

To demonstrate our model selection and testing method, we applied it to study unbiased and GC-biased acceleration in human and four nonhuman ape genomes.

We analyzed genome-wide multiple sequence alignments of 100 vertebrates and identified 272,466 mammalian conserved elements that met our stringent quality criteria (Materials and Methods). We excluded the apes we test for acceleration in defining these conserved elements. Each element was then evaluated for accelerated substitution rates in the lineages leading to human, chimpanzee, gorilla, orangutan, and gibbon from their most recent common ancestor with another ape using the model selection and testing procedure described above (fig. 1). This analysis of 1024 partially nested combinations of unbiased and/or GC-biased acceleration in any of the ape lineages identified 5916 mammalian conserved genomic elements with statistically significant evidence of accelerated substitutions in at least one ape lineage (*P* < 0.0001; LRT of the best model compared with the null model). We chose this somewhat conservative *P*-value threshold because formally controlling a multiple testing error rate in the context of our model selection procedure is not straightforward. These linARs have more substitutions than expected in various combinations of all five lineages (table 2). Most linARs are only accelerated in one lineage. Gibbon has the most linARs, with roughly similar numbers discovered in other lineages (see “Comparison of Amounts of Acceleration across Apes” and fig. 5). The human-accelerated linARs overlap previously identified HARs at different rates depending on the prior study, ranging from 27% to 63% of candidate conserved elements analyzed in both studies but much lower percentages of all elements in a given study, which is similar to rates of overlap between pairs of prior studies (supplementary text, Supplementary Material online). These differences have been attributed to studies using different data and methods (Franchini and Pollard 2017, Levchenko, Kanapin, et al. 2018).

**Table 2.** Counts of linARs with Unbiased and/or GC-Biased Acceleration in Different Combinations of Ape Lineages.

	Human*S* > 0	Human*B* > 0	Chimp*S* > 0	Chimp*B* > 0	Gorilla*S* > 0	Gorilla*B* > 0	Orang*S* > 0	Orang*B *> 0	Gibbon*S *> 0	Gibbon*B *> 0
Human *S* > 0	1298	9	257	80	260	70	310	61	343	83
Human *B* > 0		618	95	53	80	55	114	39	122	67
Chimp *S* > 0			1426	12	197	64	250	62	294	88
Chimp *B* > 0				538	89	58	103	28	127	44
Gorilla *S* > 0					1091	3	273	49	285	71
Gorilla *B* > 0						510	97	31	114	47
Orang *S* > 0							1764	18	412	110
Orang *B* > 0								380	83	34
Gibbon *S* > 0									1814	24
Gibbon *B* > 0										575

Chimp,  chimpanzee; Orang, orangutan; *S* > 0 ,  unbiased acceleration; *B *> 0, GC-biased acceleration. Only pairs of lineages are shown; a small number of linARs are accelerated in more than two lineages.

### Evidence of Biased Gene Conversion

Our statistical models include separate parameters for unbiased acceleration (consistent with positive selection or loss of constraint) and GC-biased acceleration (consistent with gBGC or selection on GC content). In each lineage, the model selection procedure directly compares the likelihood of models with either or both of these parameters. This enables us to quantify the relative rates of unbiased and GC-biased acceleration across species. All apes had many more linARs with unbiased acceleration, although GC-biased acceleration was not uncommon (26.2% of linARs overall, range 17.7%–32.3% per ape) (table 2), consistent with estimates of the prevalence of GC-biased hits in previous studies of human accelerated noncoding regions (Kostka, Hubisz, et al. 2012, Gittelman, Hun, et al. 2015). This suggests that gBGC is prevalent in apes, albeit with differences in frequency, but it is consistently less common than the combination of positive selection and loss of constraint.

### Genomic Distribution of Ape linARs

**Fig. 2.**
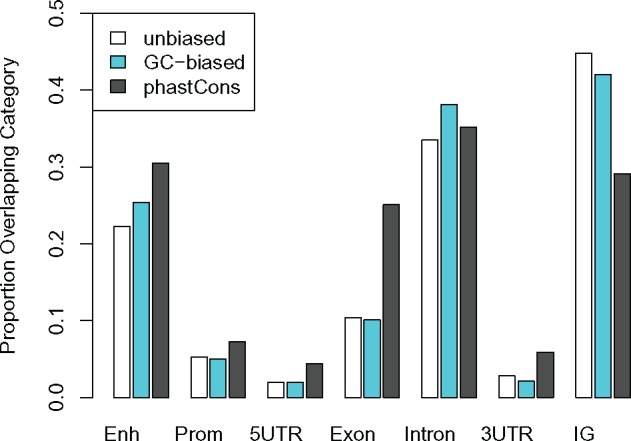
linARs are enriched in intergenic regions. For each genomic feature annotation category, bar height is the proportion of linARs (unbiased acceleration, white; GC-biased acceleration, turquoise) and all phastCons elements (gray) overlapping that feature. Enh: enhancer, Prom: promoter, 5UTR: 5′ untranslated region, 3UTR: 3′ untranslated region, IG: intergenic.

Similar to HARs, ape linARs are mostly noncoding, with their largest fraction falling in intergenic regions of the human genome (table 3). This distribution is similar to that of the phastCons elements from which linARs are drawn, except that linARs are more enriched for intergenic elements (45.6% of linAR sequence vs. 29.1% of phastCons sequence; *P*< 0.001) (fig. 2**)**. Increased enrichment for intergenic elements is consistent across linARs that are accelerated in different apes, and it holds for both unbiased and GC-biased acceleration (fig. 2, supplementary fig. S1, Supplementary Material online, table 3). Interestingly, linARs show clustering along human chromosomes, as was previously observed with HARs (supplementary fig. S2, Supplementary Material online). HARs are known to be significantly enriched at the distal ends of human chromosomes, where substitution and recombination rates are elevated (Pollard, Salama, et al. 2006, Kamm, Pisciottano, et al. 2013). But this pattern appears weaker for all ape linARs in human genome coordinates, likely due in part to chromosomal rearrangements that moved ancestrally distal chromosomal segments into nondistal regions of the human chromosomes.

**Table 3.** Proportions of the Base Pairs in linARs and All phastCons Elements That Overlap Various Genomic Features (Allowing for Multiple Overlapping Annotations) Show Enrichment of linARs in Intergenic Regions.

Model	Taxa	Enhancer	Promoter	5′UTR	Exon	Intron	3′ UTR	Intergenic	Total bp
Unbiased *S* > 0	Any species	0.223	0.053	0.020	0.104	0.335	0.028	0.448	830,741
	Human	0.219	0.027	0.004	0.045	0.361	0.016	0.482	222,423
	Chimp	0.215	0.066	0.032	0.139	0.329	0.049	0.422	210,093
	Gorilla	0.196	0.037	0.012	0.053	0.329	0.018	0.509	139,073
	Orangutan	0.235	0.057	0.018	0.108	0.317	0.017	0.438	277,151
	Gibbon	0.194	0.047	0.016	0.078	0.329	0.015	0.497	308,956
GC-biased *B* > 0	Any species	0.254	0.050	0.020	0.101	0.381	0.021	0.420	223,622
	Human	0.271	0.085	0.031	0.075	0.381	0.017	0.420	75,522
	Chimp	0.292	0.043	0.012	0.090	0.385	0.028	0.407	48,728
	Gorilla	0.284	0.045	0.005	0.077	0.367	0.037	0.460	38,524
	Orangutan	0.204	0.018	0.010	0.171	0.343	0.031	0.437	18,895
	Gibbon	0.203	0.036	0.022	0.134	0.418	0.019	0.370	56,414
Unbiased + GC-biased	Any species	0.220	0.046	0.017	0.088	0.348	0.023	0.456	1,359,183
phastCons	N/A	0.305	0.073	0.044	0.251	0.352	0.059	0.291	37,169,015

“Any species” refers one or more species.

For each species, a linAR is included in overlap if it is accelerated in only that species or multiple species (e.g., linARs with unbiased acceleration (*S* > 0) in Human that overlap enhancers could also fit a model of unbiased acceleration in Gibbon and would be included in both Unbiased Human overlap and Unbiased Gibbon overlap columns).

Unbiased + GC-biased refers to the complete set of linARs (i.e., *S *> 0 and/or *B* > 0 for any species).

phastCons refers to the base set of elements that were tested.

### Ape linARs May Function as Gene Regulatory Enhancers

The genomic distribution and evolutionary conservation (outside apes) of noncoding linARs is suggestive of regulatory function. To explore this hypothesis, we annotated phastCons elements, including linARs, with a wide variety of publicly available data, including functional genomics (ChIP-seq and RNA-seq) data and the VISTA Enhancer Browser (supplementary table S1, Supplementary Material online). We found strong support for a regulatory function for the majority of linARs, with 75.6% of linARs containing enhancer-associated marks (histone 3 lysine 27 acetylation, p300 binding) or enhancer predictions in human and/or mouse cells. This overlap with enhancer-like elements is only slightly lower than that observed for all phastCons elements (82% overlap), but this difference is statistically significant (*P* < 10^−6^). We also found that 58/117 (49.6%) linARs tested by VISTA show evidence of enhancer activity in mouse embryos, which is ∼1.26-fold more than expected given the VISTA validation rate of phastCons elements (binomial *P* = 0.0166). We hypothesize that the relative enrichment of linARs versus all phastCons elements is higher for VISTA enhancer regions compared with our compendium of enhancer-associated marks, because VISTA measures developmental enhancer activity and linARs may be preferentially active during development. Furthermore, not all regions with enhancer-associated marks are functional enhancers. In addition, since much of the enhancer annotation we analyzed is based on human sequences and/or human cells, further studies are needed to determine if the putative regulatory functions of linARs are conserved across apes.

### Developmental Loci Are Enriched for Ape linARs

Given the potential regulatory role of linARs, we were curious about the functions of genes regulated by linARs. We therefore mapped each linAR to the nearest gene and tested if genes associated with linARs are enriched for any gene ontology categories compared with phastCons elements. We found a strong enrichment for genes involved in developmental processes, in particular central nervous system development, as well as functions related to transcription factor activity (supplementary table S2, Supplementary Material online). This pattern is consistent across linARs from different apes (supplementary tables S3–S7, Supplementary Material online) and similar to the functional enrichments previously reported for HARs (Lindblad-Toh, Garber, et al. 2011), demonstrating a shared link between accelerated sequence evolution and developmental processes across apes. The enrichment of developmental regulators amongst linAR-associated genes is robust to the bioinformatics method for mapping phastCons elements to genes (Materials and Methods) (supplementary table S8, Supplementary Material online). The enrichment is weaker, however, when analyzed from the perspective of the phastCons elements (linAR vs. not) rather than the perspective of the genes (linAR-associated vs. not). This difference is driven in part by genes with large regulatory domains that harbor many phastCons elements. Thus, linARs frequently occur close together on the genome nearby developmental transcription factors, but these loci also harbor many nonaccelerated conserved noncoding sequences.

### Hotspots of Accelerated Evolution within and across Ape Species

Because linARs are clustered in the human genome (supplementary fig. S2, Supplementary Material online), we sought to identify specific genomic regions with large clusters of linARs. First, we considered each ape separately. For each species, we compared the median distance between closest linARs for that species to the same statistic computed on random sets of equal numbers of phastCons elements (Materials and Methods) (supplementary table S9, Supplementary Material online). This analysis showed that linARs are significantly more clustered than phastCons elements for each species alone: human (*P* < 0.001), chimpanzee (*P* = 0.002), orangutan (*P* < 0.001), gorilla (*P* < 0.001), and gibbon (*P* < 0.001). Since phastCons elements are themselves fairly clustered, we conclude that linARs show strong clustering in all five apes.

**Fig. 3.**
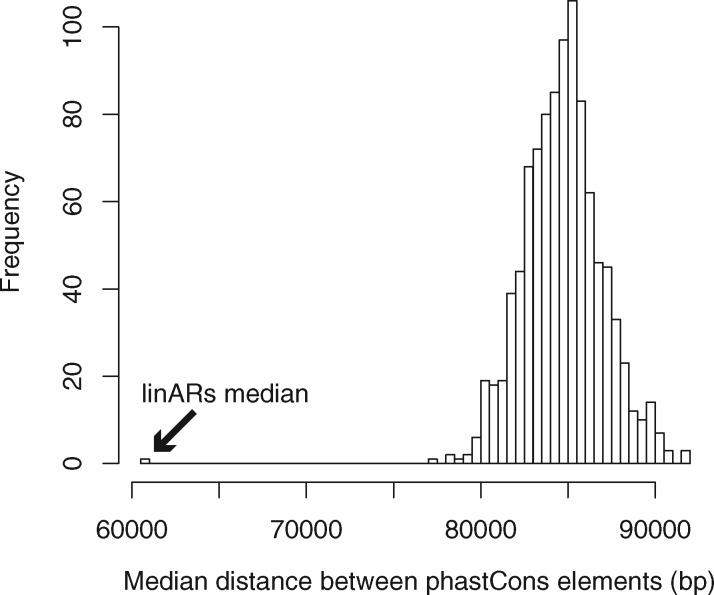
The linARs are more clustered than phastCons elements. The distribution of median distances between phastCons elements was computed using 1000 random draws of 5916 phastCons elements from the full set. The minimum sampled pairwise distance between phastCons elements (∼77 kb) was much larger than the median distance between pairs of linARs (60.8 kb; arrow). This analysis shows that linARs are significantly closer to each other in the human genome than are random sets of the same number of phastCons elements (*P* < 0.001).

We next sought to compare the genomic distribution of linARs across species. Using human genome coordinates, we repeated the statistical test for distance to the nearest linAR including all 5916 linARs. This revealed that linARs are closer together on average than expected given the genome-wide distribution of phastCons elements (*P* < 0.001) (fig. 3). To identify discrete linAR clusters, we first clustered phastCons elements into groups for which the longest distance between consecutive elements is less than 100 kb. We found 175 (out of 1164) such clusters that contain more linARs than expected [false discovery rate (FDR) < 0.05; binomial test] (supplementary table S10, Supplementary Material online). Most clusters contain linARs from multiple different apes. Furthermore, all clusters are located in syntenic regions of the other ape genomes with their clustering preserved (Materials and Methods). Thus, not only do ape linARs cluster within species, but these clusters are also frequently maintained across species.

**Fig. 4.**
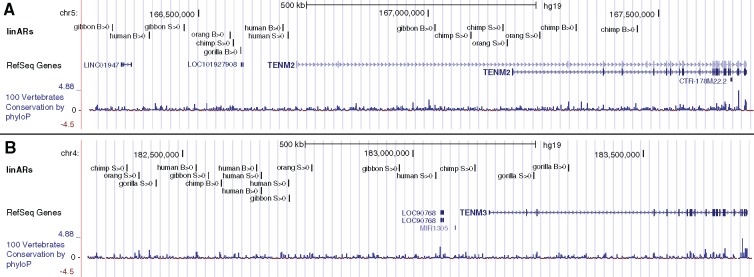
Clusters of linARs nearby teneurin transmembrane genes. Browser views of loci containing two of the largest multispecies linAR clusters nearby the genes (*A*) *TENM2* and (*B*) *TENM3*. Genes and conservation are shown with UCSC Genome Browser tracks. A custom track shows linARs annotated by species and unbiased acceleration (*S* > 0) or GC-biased acceleration (*B* > 0).

To explore the set of genes nearby linAR clusters, we mapped linAR clusters to any gene within the cluster boundaries or the closest gene if the cluster is intergenic and then ranked the resulting 219 genes based on the size of their clusters (supplementary table S10, Supplementary Material online). Many of the top genes are developmental transcription factors and signaling proteins, including many expressed during development of the central nervous system and sensory organs. For example, one hotspot for linARs is a group of four clusters comprising 42 linARs that are located nearby each other in the locus of *ROBO1* and *ROBO2*, transmembrane genes that function as receptors for SLIT family proteins in axon guidance and cell migration. We also identified multispecies linAR clusters in the FOXP1 and FOXP2 loci. The largest cluster is a nearly 1.5-mb region on human chromosome 4 with 62 linARs, including elements accelerated in each ape. A potential gene target for these linARs is the neurodevelopmental regulator *TENM3* (fig. 4*B*)*.* Supporting the hypothesis that regulation of *TENM3* evolved rapidly in different ape lineages, another large cluster of mixed-species linARs is located nearby *TENM2* (fig. 4*A*)*.* These teneurin transmembrane proteins are coexpressed in neurodevelopment and can form a heterodimer. Finally, our analysis found a cluster of 36 linARs from multiple apes nearby the *NPAS3* gene, which was previously shown to harbor a cluster of HARs, several of which are validated neurodevelopmental enhancers (Kamm, Pisciottano, et al. 2013). Together these results show that a discrete set of developmental regulatory loci have been subject to accelerated evolution in multiple ape lineages.

### Comparison of Amounts of Acceleration across Apes

**Fig. 5.**
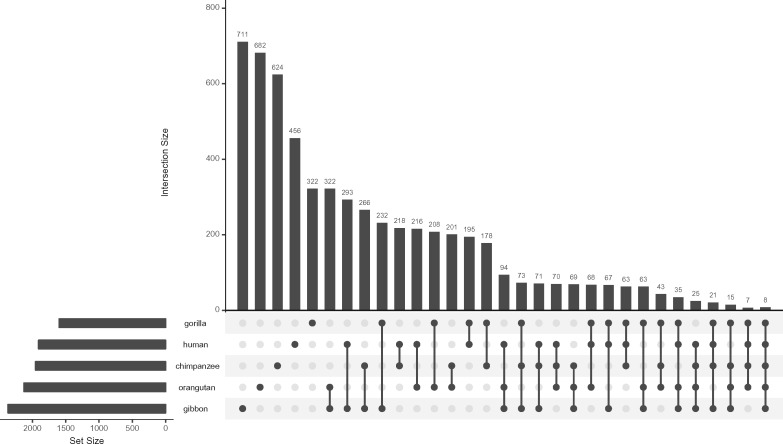
Apes have similar numbers of linARs. The upset plot (histogram) of linARs with acceleration on different subsets of lineages reveals three patterns: (1) most linARs are accelerated in one or two lineages, (2) genomes with lower quality assemblies (e.g., gibbon) have the most linARs alone and in combination with acceleration in other lineages, and (3) species otherwise have similar number of linARs alone and in combination with each of the other apes. The horizontal bars at the left show the total number of linARs for each species, whereas the vertical bars show the number of linARs in each set (acceleration in single species and combinations of multiple species) as denoted by the connected circles below the histogram.

Our analyses in principle enable a direct comparison of the number of linARs across species. This comparison is confounded, however, by differences in the quality of genomes in the multiple sequence alignments we analyzed, including differences in sequence depth, assembly errors, and alignment artifacts, as well as the fact that the human assembly was used to scaffold some other genome assemblies. Supporting this confounding, the number of linARs with acceleration is negatively correlated with genome coverage, being lowest for human and gorilla. Thus, although there are different total numbers of linARs across the five ape species (horizontal bars in fig. 5), we are cautious about ascribing this to differences in evolutionary pressures. We therefore conclude that no ape genome, including the human genome, has strong evidence for a rate of lineage-specific selection or loss of constraint that is particularly high compared with others.

Because we evaluated models with combinations of unbiased and GC-biased acceleration in multiple lineages, we could further evaluate patterns of co-occurrence of linARs on the ape phylogeny (fig. 5, table 2). Most linARs are accelerated in only one ape, with acceleration in two apes being next most common. Only 111 linARs have evidence of acceleration in four or five of the apes. Nonetheless, all pairs of apes share more linARs than expected by chance (FDR < 0.05). The rate of shared linARs is about twice as high for unbiased acceleration (∼8%) compared with GC-biased acceleration (∼4%), perhaps reflecting recurrent selection, recurrent loss of constraint, and/or lineage-specific recombination hotspots. These could also reflect acceleration on an ancestral branch (e.g., loss of constraint in the common ancestor), even when other apes in the clade are not identified as accelerated, which could be false negatives. Amongst the linARs with evidence of acceleration in two or three ape genomes, one of the species is commonly gibbon, which has the most linARs and hence a higher probability of them overlapping with other species. After adjusting for the number of unbiased and GC-biased linARs per species, we observed no phylogenetic signal in the amount of sharing of linARs between ape species, except that sister species have slightly lower rates of sharing of unbiased (but not GC-biased) linARs than more distantly related species pairs. Based on our simulations, we may not have had high power to detect a weak phylogenetic signal in the amount of sharing of linARs. These results suggest that the evolutionary forces that accelerated the evolution of linARs typically affected just one lineage. In some genomic loci, however, we find evidence that these forces acted recurrently during ape evolution, as has been observed in analyses of great ape population genetic diversity (Cagan, Theunert, et al. 2016) and incomplete lineage sorting (Munch, Nam, et al. 2016).

## Discussion

We developed a model selection procedure to scan ape genomes in parallel for conserved elements similar to HARs. A strength of this approach is that a single set of candidate accelerated regions (e.g., phastCons elements) is analyzed with a consistent definition of unbiased and GC-biased acceleration across all the evaluated species, which make the resulting rates and patterns of accelerated evolution comparable. The model selection procedure can identify acceleration on more than one lineage and thereby enables recurrently evolving regions to be identified. Our simulations showed that the method controls false positives and has reasonable power on the ape phylogeny. Occasional errors include annotating acceleration to the wrong branch. Performance on larger trees or more diverged lineages has not been evaluated. The procedure is not specifically designed to identify acceleration on ancestral lineages or in one clade compared with another. These tests were previously implemented in the PHAST package, and should be used for such applications. It is also important to keep in mind that our model selection procedure is a forward selection algorithm that recursively adds accelerated branches to the null model and stops when no significant improvement in fit is observed, so it will tend to identify simple models that are consistent with the data.

We note that our method does not perform explicit hypothesis testing against an extended null hypothesis of neutral evolution that includes drift and gBGC (Galtier and Duret 2007). Rather, our procedure performs LRTs and annotates alignments with a final *P*-value quantifying evidence in favor of an alternative model that includes some form of lineage-specific acceleration (GC biased or unbiased) in substitution rate compared with a null model without acceleration. This means that linARs may be identified because an alternative model of with gBGC (i.e., *B* > 0) on a particular lineage fits the data better than the null model of no acceleration. Similarly, linARs may have evidence of relaxation of constraint (i.e., *S* > 0 but not accelerated more than the local neutral rate). Both of these scenarios represent neutral evolution. In other words, the null model is not the neutral model. It is possible to perform tests against an extended null hypothesis including gBGC and relaxation of constraint (e.g., Kostka, Hubisz, et al. 2012), but we did not do so for this genome-wide screen due to complications with estimating neutral substitution rates and structuring the null hypothesis to include gBGC. For a subset of linARs, it may be insightful to perform tests with custom-tailored approaches to rule out gBGC or relaxation of constraint in order to identify genomic elements more consistent with positive selection.

We applied our new approach to study accelerated evolution across the genomes of apes. These analyses revealed that the human genome is not unique in having linARs, nor in how many linARs it has. Comparing patterns across five apes, we found fairly consistent numbers of linARs. Differences in counts of linARs (range 1601–2389) may be due to variation in genome assembly quality. Another bias to consider in interpreting these results is the fact that the human genome was used as the reference genome in our analyses and was also employed in the assembly of other ape genomes. Across species, we also found fairly similar proportions of unbiased versus GC-biased linARs (mean 26.2%). Variation in the GC-biased proportion (range 17.7%–-32.2%) could potentially reflect differences in rates and patterns of gBGC versus positive selection or loss of constraint between apes.

Another striking similarity of linARs across apes is that they cluster together (64% within 100 kb of another linAR), both within and between species, in loci harboring developmental genes (enrichment = 1.1, FDR < 0.0004). These are mostly distinct noncoding elements in the same locus, though some linARs are accelerated in two or more of the ape lineages. An intriguing question for future research is to determine the mechanisms driving the clustering of linARs. Here we analyzed linARs in comparison to phastCons clusters in the human genome (reference sequence in alignments) and eliminated the possibility that linARs clustering is simply due to the higher number of conserved elements in developmental loci or the larger intergenic distances in these loci, which results in more conserved elements being closest to a developmental gene. Although the full set of conserved elements did show clustering, linARs were more densely clustered than other conserved elements and more frequently associated with developmental loci. One possibility is that recurrent selection on the expression levels of certain developmental genes has occurred throughout primate evolution, which is consistent with other studies that found evidence of recurrent selection in the ape species we analyzed (Cagan, Theunert, et al. 2016, Munch, Nam, et al. 2016). Alternatively, certain genes may tolerate more regulatory evolution [though developmental gene expression tends to be deeply conserved (Li, Huang, et al. 2014)]. Perhaps the simplest explanation for clusters of linARs across species is that the genomic regions containing linARs have been subject to recurrent loss of constraint. This could be due to particularly redundant enhancers that can be lost or changed with limited effects on gene expression or due to higher mutation rates in these regions. Previous work found no evidence in support of elevated mutation rates in 40-kb regions nearby 49 HARs (Katzman, Kern, et al. 2010), but future studies are needed to confirm if this is true for linARs more broadly and to what extent loss of constraint can explain clustering of linARs.

Ape linARs are particularly enriched nearby developmental transcription factors and other regulators of embryonic development, as was previously observed for HARs (Lindblad-Toh, Garber, et al. 2011). This suggests the tantalizing hypothesis that mutations in linARs altered morphogenesis during ape evolution by modifying expression levels of key regulators of embryonic development. In this context, we note that linARs are enriched for VISTA developmental enhancers compared with phastCons elements but depleted for enhancer-associated marks in general (see Results). This difference may be biological (i.e., due to linARs being developmental enhancers) but could also be explained by confounding factors, including enhancer marks not being perfect proxies for enhancer function and also differing between individuals. Notably our analyses of both VISTA and a compendium of enhancer-associated marks find evidence that a large fraction of linARs may function as enhancers.

In future work, it would be interesting to test if lineage-specific changes in the sequences of nonhuman ape linARs alter gene expression during embryonic development, as has been demonstrated for many HARs (Hubisz and Pollard 2014, Boyd, Skove, et al. 2015). If the linARs that cluster nearby a developmental regulator are enhancers that control distinct spatial or temporal aspects of that gene’s expression, then one could hypothesize that their accelerated evolution in different apes might be associated with morphological features that diverged during ape evolution. On the other hand, the multiple regulatory elements nearby a developmental gene may also buffer or otherwise affect each other (Long, Prescott, et al. 2016), making it hard to predict the effect that sequence changes in one small noncoding element will have on gene expression and organismal phenotypes. These questions will be best answered using functional studies that test linARs not only individually, but also collectively. These investigations may soon be possible with high-throughput technologies, such as genome editing and massively parallel reporter assays, which enable thousands of regulatory elements to be investigated en masse in primate cells. With these approaches, the role of linARs in primate evolution and their clustering in specific developmental pathways could soon be elucidated.

## Materials and Methods

Additional details about methods are available in the supplementary text, Supplementary Material online. In addition to providing open source software (linACC), we posted most of our analysis scripts plus accompanying data sets online at: http://www.kostkalab.net/pub_software/linACC/supplement/linACC_supplement.html, last accessed June 1, 2018.

### Sequence Data

We obtained **multiz** whole-genome sequence alignments of 100 vertebrates and associated phylogenetic trees for the autosomes and chromosome X from the UCSC Genome Browser (http://hgdownload.soe.ucsc.edu/goldenPath/hg19/multiz100way/, last accessed June 1, 2018). The reference genome for the alignments is human (hg19 assembly).

### Conservation

To analyze a consistent set of genomic elements that are likely functional, we used the **phastCons** program to identify mammalian conserved elements genome-wide whereas excluding all apes from the computations (human reference sequence masked and other apes dropped from the alignments). Command line parameters were: –rho 0.3 –expected-length 45 –target-coverage 0.3. These are the standard parameters used in the UCSC Genome Browser protocol, and we used the phylogenetic model from the UCSC conserved elements track providing branch lengths of the phylogenetic tree, background frequencies, and parameterizations for a general reversible substitution rate matrix. The genome was analyzed in 10 megabase (Mb) blocks to facilitate computations. Conserved elements separated by less than 10 bp were merged, and then any elements shorter than 50 bp were dropped, since power to detect ape acceleration is low on short alignments (Pollard, Hubisz, et al. 2010). We also dropped any element where any of the five apes (human, chimpanzee, gorilla, orangutan, and gibbon) was not present in the alignment (i.e., at least one site with a nucleotide). This produces 660,077 conserved elements that cover 94,370,847 bp of the human genome. Alignments corresponding to each conserved element were extracted from the 100-species genome-wide alignments.

### Filtering

To minimize the influence of sequencing, assembly, and alignment errors on our inferences regarding accelerated substitution rates, we filtered the conserved element alignments using an extension of a previous approach (Lindblad-Toh, Garber, et al. 2011). We first masked repeat sequences (UCSC srpt and rmsk tracks) annotated in each of the five ape genomes that we tested for accelerated evolution (hg19, panTro4, gorGor3, ponAbe2, nomLeu3). Next, we generated genome-wide self-alignments following the UCSC selfChain documentation: align to self using **lastz**, and then chain into longer contiguous alignments. Bases in these self-similar regions were masked (replaced with Ns) in the conserved element alignments to avoid false inferences of acceleration due to misaligned repeats or structural variants. We then dropped alignments corresponding to annotated pseudogenes (pseudoYale60 UCSC Genome Browser track), segmental duplications (genomicSuperDups track), and self-similar genomic regions (selfChain, see above) annotated on the reference genome (hg19). Finally, we used the UCSC netSyntenic tracks to require that conserved elements fall within blocks of level-1 or level-2 nongapped synteny between human (hg19) and (1) macaque (rheMac3), (2) dog (canFam3), and (3) mouse (mm10). Together, these conservative filters likely remove some truly accelerated elements, but they are necessary to avoid thousands of false inferences of accelerated evolution due to misaligned paralogous sequences and other errors that are present in genome-wide alignments (Pollard, Salama, et al. 2006, Lindblad-Toh, Garber, et al. 2011). This bioinformatics pipeline generated multiple sequence alignments for 272,466 conserved elements (“phastCons elements”) covering 37,152,199 bp of the human genome that are our candidates for accelerated evolution in apes.

### Testing for Acceleration in Ape Lineages

We applied our model selection procedure (see above) to multiple sequence alignments of the 272,466 high-quality phastCons elements to test for accelerated evolution in five apes: human, chimpanzee, gorilla, orangutan, and gibbon. For five lineages, there are 2^2^^×^^5^ = 1024 different models per alignment, which we screened with our forward selection algorithm. To implement the forward selection procedure, we used the RPHAST package (Hubisz, Pollard, et al. 2011) for model fitting and calculated LRT *P*-values using the asymptotic distribution of the LRT statistic for models differing in one nonnegative parameter (Self and Liang 1987). As a null model we used the model from the phastCons analysis (see paragraph “conservation” above), except that background frequencies were adapted to reflect the local GC-content of the alignment, excluding primates. This modification is necessary because otherwise local changes in GC-content could lead to the spurious annotation of GC-biased substitution rate increases. The only free parameter of the null model is the overall tree scale (relative branch lengths were kept constant), which allows the model to adjust to changes in the mutation rate across the genome. Alternative models additionally have a second parameter encoding a different rate of substitutions on the target branch or set of branches. In our model selection procedure, we perform LRTs between nested models that differ in exactly one parameter (see above), and the test is determining if this extra parameter significantly improves the likelihood. We chose the *P*-value cut-off *P*_cut_ to be 0.01 for these LRTs. After model selection, we ultimately annotate each alignment with the null model or with the most complex alternative model with *P*_cut_ < 0.01. If the annotated model was different from the null model, we performed an additional final LRT comparing the annotated model with the null model. We note that this comparison has not been performed in the model selection procedure if the annotated model has more than one extra free parameter compared with the null model (i.e., *P*_cut_ for the annotated model compares it with a nested model that itself fit better than the null model). This final *P*-value summarizes the support in the alignment data of the annotated alternative model compared with the null model, and we used it prioritize 5916 lineage specific accelerated regions across the genome by choosing a cut-off of *P* < 0.0001.

### Annotation

We explored the genomic distribution and potential functions of the resulting ape linARs using the UCSC known genes annotation (http://genome.ucsc.edu, last accessed June 1, 2018), the VISTA Enhancer Browser (http://enhancer.lbl.gov, last accessed June 1, 2018), ChromHMM genome segmentations of ENCODE data (https://www.encodeproject.org, last accessed June 1, 2018), FANTOM5 enhancers (http://fantom.gsc.riken.jp/5/, last accessed June 1, 2018), and other functional genomics data from publicly available databases. We defined enhancer as any of the following annotations (see supplementary table S1, Supplementary Material online for definitions and references): chromHMMenh, encodeDNaseIHS, encodeH3K27ac, ORegAnno_hg19, encodeP300, p300Shen, p300Blow, Fantom5, vistaEnhVisel, enhRada. We defined promoter with the single annotation chromHMMprom, and we define gene features using the UCSC hg19 “known gene” annotation merging across genes to allow overlapping gene features but preventing double counting a feature present in two overlapping genes. For example, a region in exons of two genes is counted once as an exon and a region in an exon of one gene plus an intron of another gene is counted once as an exon and once as an intron. Each phastCons element (linARs and non-linARs) was annotated with all overlapping data, and annotation patterns were compared between linARs and all phastCons elements.

### Ontology

To test if linARs are preferentially associated with particular genes, we mapped each phastCons element (linARs and non-linARs) to the closest gene. These closest genes were used to compare gene ontology categories for genes associated with linARs versus genes associated with a phastCons element using GOrilla (Eden, Lipson, et al. 2007, Eden, Navon, et al. 2009) (http://cbl-gorilla.cs.technion.ac.il, last accessed June 1, 2018) with default settings. Genes associated with phastCons elements were identified using a random subset of 20,000 elements for computational efficiency. We report FDR and enrichment statistics for all GO terms with FDR < 0.1. We also used GREAT (McLean, Bristor, et al. 2010) (http://bejerano.stanford.edu/great/public/html/, last accessed June 1, 2018) to perform enrichment analyses that are noncoding element based, rather than gene based, and therefore account for the unequal size of regulatory domains of genes. GREAT associates noncoding regions with genes using genomic proximity, transfers ontology terms from genes to their associated noncoding regions, and then tests for enrichment in the two-by-two table counting noncoding regions with and without an ontology term and with or without being a linAR. To assess robustness, enrichment analyses were repeated using other methods of mapping phastCons elements to genes: two nearest genes and the basal-plus-extension method as implemented in GREAT (supplementary table S8, Supplementary Material online). For basal-plus-extension method, we used the following parameters: Each gene is assigned a basal regulatory domain of 5-kb upstream and 1-kb downstream of the TSS (regardless of other nearby genes). The gene regulatory domain is extended in both directions to the nearest gene's basal domain but no more than 1 Mb in one direction.

### Clustering

We investigated whether linARs within and across apes occur closer together along the human genome than expected given the density of phastCons elements. For each linAR, we computed the genomic distance to the nearest other linAR. Then we did the same for all phastCons elements. To test if the median distance between linARs is shorter than expected given the distances between phastCons elements, we randomly sampled (without replacement) 1000 sets of phastCons elements of the same size as the number of linARs and computed the median distance between phastCons elements for each set. The proportion of these median distances that exceeded the median distance between linARs is the empirical *P*-value.

As a second approach, we identified clusters of phastCons elements where consecutive elements are separated by no more than 100 kilobases (kb) in the human genome (hg19, reference sequence in alignments). For each cluster containing at least one linAR, we calculated the number *n* of phastCons elements and the number *k* of those that are linARs. We then computed a Binomial *P*-value Bin(*k* | *p*, *n*) for the cluster containing *k* linARs out of *n* phastCons elements, under the null hypothesis that linARs are not clustered any more than typical phastCons elements (i.e., the probability *P* of a phastCons element in the cluster being a linAR was set equal to the overall proportion of linARs among phastCons elements genome wide). We accounted for the fact that each cluster has at least one linAR by dividing the resulting *P*-values by 1-Bin(0 | *p*, *n*) before performing multiple testing correction to control the FDR (Hochberg and Benjamini 1990). This enrichment test yielded a set of clusters with more linARs than expected.

To evaluate synteny of linAR clusters in the nonhuman ape genomes, we used UCSC level 1 and 2 syntenic net tables (hg19 assembly).

## Supplementary Material

Supplementary data are available at *Molecular Biology and Evolution* online.

## Supplementary Material

Supplementary Data
