# Supplementary material for: Developmental Loci Harbor Clusters of Accelerated Regions That Evolved Independently in Ape Lineages
Source: Mol Biol Evol. 2018 Jun 18;35(8):2034–45. doi: 10.1093/molbev/msy109 (PMC6063267; doi:10.1093/molbev/msy109)
Supplement: Supplementary Data [file msy109_supp.zip › linARs-Supplement_r2.docx]

**SUPPLEMENTARY MATERIAL**

**Developmental loci harbor clusters of accelerated regions that evolved independently in ape lineages**

Dennis Kostka^1^, Alisha K. Holloway^2,3,4^, Katherine S. Pollard^2,4*^

1: Departments of Developmental Biology and Computational & Systems Biology, University of Pittsburgh School of Medicine, 530 45th Street, Pittsburgh, PA 15201

2: Gladstone Institutes, 1650 Owens Street, San Francisco, CA 94158

3: Phylos Bioscience, Portland, OR 97201

4: Division of Bioinformatics, Institute for Human Genetics and Institute for Computational Health Sciences, University of California, San Francisco, CA 94158

* *Author for Correspondence*: Katherine S. Pollard, Gladstone Institutes, San Francisco, CA 94158, 415-734-2711, kpollard@gladstone.ucsf.edu

**SUPPLEMENTARY TEXT**

**Mammalian conserved elements without primate sequences**

To generate conserved regions across mammals excluding the primates we analyze for lineage specific acceleration, we proceeded as follows: We downloaded hg19-based 100way alignments form UCSC (<http://genome.ucsc.edu>), and the phylogenetic model (hg19.100way.phastCons.mod)  used for the corresponding phastCons (“most conserved elements”) track, also available there. Non-mammalian vertebrate species were pruned from the model. Then, 100way alignments split into pieces of ~10mb size with the human sequence masked, and with chimpanzee, gorilla, orangutan, gibbon and non-mammalian vertebrates removed, since we aim to detect acceleration on these lineages. The phastCons program (<http://compgen.cshl.edu/phast>) was run on these alignments in 1-mb chunks, with the phylogenetic model obtained as described above, except that equilibrium base frequencies were adjusted to reflect the average GC-content of each 10mb region. The phastCons parameters used were the standard ones from the UCSC phastCons protocol:

       --rho 0.3

       --expected-length 45

       --target-coverage 0.3

       --most-conserved

       --score

Results for each chromosome were aggregated into a bed file containing the conserved regions for further analysis. Of these mammalian phastCons elements (with apes dropped from the scoring procedure), 92% overlap the “regular” 100way phastCons elements from UCSC (derived from alignments including the apes we dropped as well as some non-mammal vertebrates). On average 86% of bases in each of our phastCons elements are covered by the “regular” elements.

This analysis was implemented using custom scripts, and a bed file containing the mammalian “non-primate” conserved regions is available as supplemental data.

**A set of candidate alignments for lineage-specific acceleration**

Based on these phastCons elements, we generated alignments that were further analyzed for lineage specific acceleration. First, we merged conserved regions that were less than 10bp apart and dropped regions that were less than 50bp long. These regions were the filtered with a strict set of criteria [1]: level 1 or level 2 non-gap synteny between human and all of macaque, mouse, and dog (netSynteny); no pseudogenes (luNega and pseudoYale); no segmental duplications (genomicSupDups); no repeat elements (rmsk); and no human paralogs (selfChain). Next, self-alignments were newly generated for all five primates, and repeat information was obtained from UCSC (RepeatMasker tracks).  Alignment blocks covering the (filtered) non-mammalian conserved regions were extracted from the UCSC 100-way alignments, and repeats and pseudo-alignments were masked in each primate. These alignments blocks were then used as input for our model selection procedure. These analyses were implemented with custom scripts.

**Comparison of human lineage-specific accelerated regions with previously annotated loci**

We compared the set of linARs with acceleration in the human lineage to previously annotated loci with human-specific evolutionary signatures [1-4]. We first constrained ourselves to the non-coding part of the genome since many prior studies of human-acceleration did so, and then we counted human linARs (hg-linARs) that overlap with other human-accelerated regions. We defined region A is overlapped by region B if region B covers at least 50% of A’s bases.

| **Study** | **# regions** | **# regions in this study** | **# regions overlapped by hg-linARs** | **# hg-linARs overlapped by regions** |
| --- | --- | --- | --- | --- |
| **HARs [2]** | **189** | **123** | **53** | **41** |
| **2xHARs**  **[1]** | **507** | **280** | **177** | **135** |
| **ANC**  **[3]** | **1,321** | **496** | **136** | **149** |
| **HACNS**  **[4]** | **985** | **499** | **174** | **182** |

The linARs with acceleration in human overlap the other sets as much as we have come to expect from analyzing similarity between these other sets of regions [5]. For instance, contrasting both ANC and hg-linARs against HARs, 2xHARs and HACNS, we find for the fractions of ANC and hg-linARs covered, respectively, we find that the fraction of ANC and hg-linARs that are overlapped by other regions are comparable.

| **Study** | **Number of regions** | **Fraction overlapped by ANC** | **Fraction overlapped by hg-linARs** |
| --- | --- | --- | --- |
| **HARs**  **[2]** | **189** | **0.19** | **0.28** |
| **2xHARs**  **[1]** | **507** | **0.16** | **0.35** |
| **HACNS**  **[4]** | **985** | **0.16** | **0.18** |

**REFERENCES**

**[1]** Lindblad-Toh K, Garber M, Zuk O, Lin MF, Parker BJ, Washietl S, Kheradpour P, Ernst J, Jordan G, Mauceli E and others. A high-resolution map of human evolutionary constraint using 29 mammals. Nature 2011;478(7370):476-82.

**[2]** Pollard KS, Salama SR, King B, Kern AD, Dreszer T, Katzman S, Siepel A, Pedersen JS, Bejerano G, Baertsch R and others. Forces shaping the fastest evolving regions in the human genome. PLoS Genet 2006;2(10):e168.

**[3]** Bush EC, Lahn BT. A genome-wide screen for noncoding elements important in primate evolution. BMC Evol Biol 2008;8:17.

**[4]** Prabhakar S, Noonan JP, Paabo S, Rubin EM. Accelerated evolution of conserved noncoding sequences in humans. Science 2006;314(5800):786.

**[5]** Franchini LF, Pollard KS. Human evolution: the non-coding revolution. BMC Biol 2017;15(1):89.

**SUPPLEMENTARY FIGURES**

**
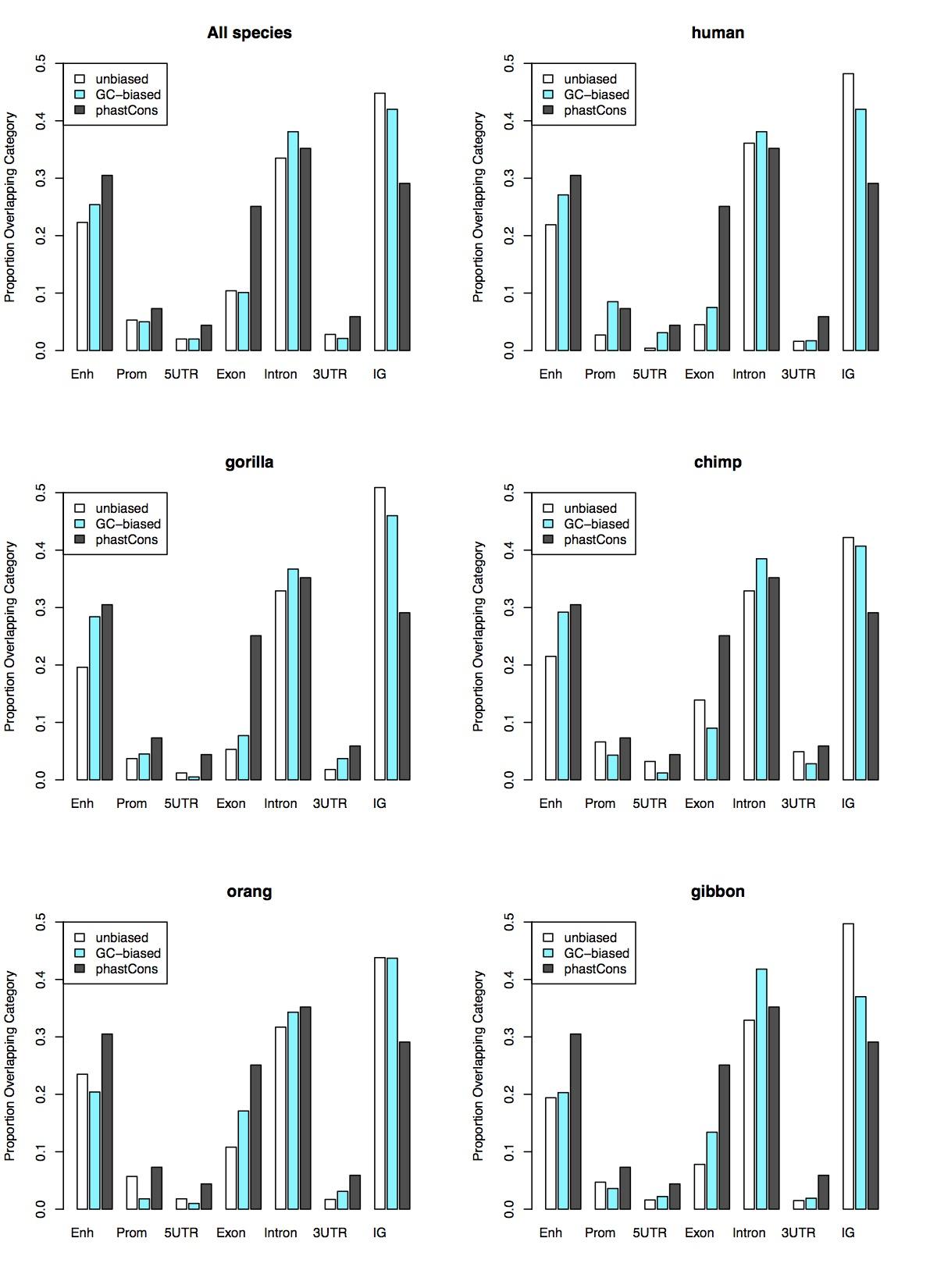
**

**Figure S1 – linARs are enriched in intergenic regions across primates.** Proportions of linARs with unbiased acceleration (S>0) or GC-biased acceleration (B>0) that overlap different genomic annotation categories are shown next to the background proportions for phastCons elements. Separate panels show results using linARs specific to each ape species. Annotations are from the human genome assembly. Panel “All species” (i.e., accelerated in any species) is the same as Figure 2. Data is from Table 1.

**
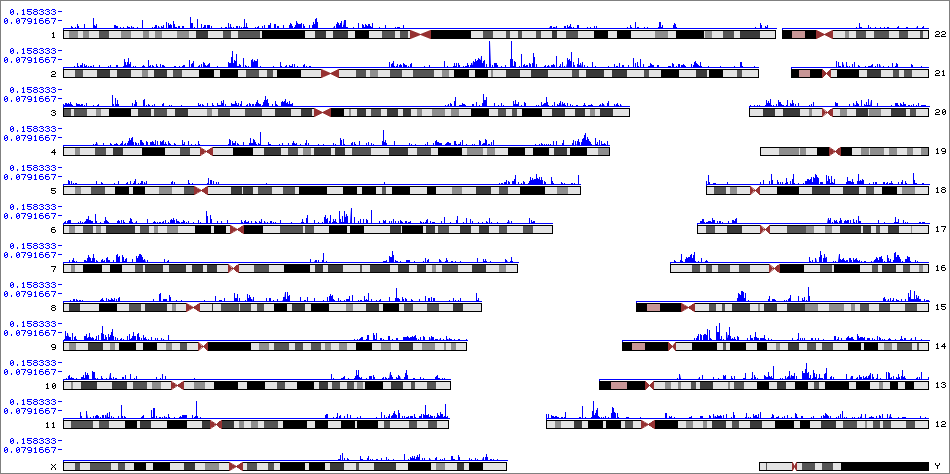
**

**Figure S2 – linARs are clustered in the human genome.** The density of linARs (blue) is plotted on a genome graph of the human chromosomes. There are genomic regions with dense clusters of linARs.

**SUPPLEMENTARY TABLES**

*Separate Files.*

**Table S1 – Functional genomics annotations of linARs.**

**Table** **S2 – Gene Ontology (GO) enrichments for nearest genes to linARs.** Analyses performed with GOrilla. Genes are annotated as being the closest TSS to a linAR or not and having each annotation or not. The background is based on mapping phastCons elements nearest genes instead of linARs. GO terms with FDR < 0.1 are shown.

**Table** **S3 – Gene Ontology biological process enrichments for nearest genes to human linARs.** Genes are annotated as being the closest TSS to a human accelerated linAR or not and having each annotation or not. The background is based on mapping phastCons elements nearest genes instead of linARs. GO terms with FDR < 0.1 are shown.

**Table** **S4 – Gene Ontology biological process enrichments for nearest genes to chimpanzee linARs.** Genes are annotated as being the closest TSS to a chimpanzee accelerated linAR or not and having each annotation or not. The background is based on mapping phastCons elements nearest genes instead of linARs. GO terms with FDR < 0.1 are shown.

**Table** **S5 – Gene Ontology biological process enrichments for nearest genes to gorilla linARs.** Genes are annotated as being the closest TSS to a gorilla accelerated linAR or not and having each annotation or not. The background is based on mapping phastCons elements nearest genes instead of linARs. GO terms with FDR < 0.1 are shown.

**Table** **S6 – Gene Ontology biological process enrichments for nearest genes to orangutan linARs.** Genes are annotated as being the closest TSS to a orangutan accelerated linAR or not and having each annotation or not. The background is based on mapping phastCons elements nearest genes instead of linARs. GO terms with FDR < 0.1 are shown.

**Table** **S7 – Gene Ontology biological process enrichments for nearest genes to gibbon linARs.** Genes are annotated as being the closest TSS to a gibbon accelerated linAR or not and having each annotation or not. The background is based on mapping phastCons elements nearest genes instead of linARs. GO terms with FDR < 0.1 are shown.

**Table S8 – GO biological process enrichments for nearest genes to linARs using basal-plus-extension mapping to genes.** Analyses performed with GOrilla. Genes are annotated as being associated with a linAR or not using the basal-plus-extension method (see Methods) and having each annotation or not. The background is based on mapping phastCons elements genes (also with basal-plus-extension mapping) instead of linARs. These can be compared to the biological process results in Table S2, which are for the same linARs but using closest-gene mapping rather than basal-plus-extension. GO terms with FDR < 0.1 are shown.

**Table** **S9 – Table S9. Shorter median distance between species-specific linARs than expected compared to phastCons elements.**

**Table** **S10 – Multi-species clusters of linARs.** Clustering of linARs (all species combined) was performed using human genome (hg19) coordinates (see Methods). All clusters contain significantly more linARs than expected given the genome-wide distribution of phastCons elements.
