# Supplementary material for: Developmental Loci Harbor Clusters of Accelerated Regions That Evolved Independently in Ape Lineages
Source: Mol Biol Evol. 2018 Jun 18;35(8):2034–45. doi: 10.1093/molbev/msy109 (PMC6063267; doi:10.1093/molbev/msy109)
Supplement: Supplementary Data [file msy109_supp.zip › TableS9.docx]

|  | **Median distance between species-specific linARs (bp)** | **Mimimum* median distance between phastCons elements (bp)** |
| --- | --- | --- |
| Human | 237,228 | 393,770 |
| Chimp | 409,228 | 413,822 |
| Gorilla | 592,681 | 601,826 |
| Orangutan | 303,911 | 362,463 |
| Gibbon | 223,773 | 289,698 |

**Table S9. Shorter median distance between species-specific linARs than expected compared to phastCons elements.** *Minimum is over 1,000 draws of the same number of phastCons elements as linARs for each species.
